# The Endothelial ADMA/NO Pathway in Hypoxia-Related Chronic Respiratory Diseases

**DOI:** 10.1155/2014/501612

**Published:** 2014-02-25

**Authors:** Nicole Lüneburg, Lars Harbaum, Jan K. Hennigs

**Affiliations:** ^1^University Medical Center Hamburg-Eppendorf, Department of Clinical Pharmacology and Toxicology, Martinistraße 52, 20246 Hamburg, Germany; ^2^University Medical Center Hamburg-Eppendorf, II Department of Medicine-Oncology, Hematology, Stem Cell Transplantation, Section of Pneumology, Hamburg, Germany; ^3^The Vera Moulton Wall Center for Pulmonary Vascular Disease and Cardiovascular Institute, Stanford University - School of Medicine, Stanford, USA

## Abstract

Since its discovery, many adhere to the view that asymmetric dimethylarginine (ADMA), as an inhibitor of the synthesis of nitric oxide (NO), contributes to the pathogenesis of various diseases. Particularly, this is evident in disease of the cardiovascular system, in which endothelial dysfunction results in an imbalance between vasoconstriction and vasodilatation. Even if increased ADMA concentrations are closely related to an endothelial dysfunction, several studies pointed to a potential beneficial effect of ADMA, mainly in the context of angioproliferative disease such as cancer and fibrosis. Antiproliferative properties of ADMA independent of NO have been identified in this context. In particular, the regulation of ADMA by its degrading enzyme dimethylarginine dimethylaminohydrolase (DDAH) is the object of many studies. DDAH is discussed as a promising therapeutic target for the indirect regulation of NO. In hypoxia-related chronic respiratory diseases, this controversy discussion of ADMA and DDAH is particularly evident and is therefore subject of this review.

## 1. Introduction: The Endothelial ADMA/NO Pathway

### 1.1. Nitric Oxide

Endothelial-derived NO is known to be the major mediator regulating vasomotor tone. NO is involved in a wide range of mechanisms with regulatory function, including inhibition of platelet adhesion and aggregation, of monocyte adhesion and of smooth muscle cell proliferation. In this way, NO plays a crucial role in vascular homeostasis. NO is produced by nitric oxide synthase (NOS) enzymes [1]. There are three distinct isoforms which catalyze the formation of NO from the substrate L-arginine and O_2_ with L-citrulline being produced as a second product. The distinct isoforms differ in their tissue and cell type distribution as well as in their regulatory mechanisms [2]. The three isoforms are neuronal NOS (NOS1, nNOS) [3], inducible NOS (NOS2, iNOS) [4], and the endothelial NOS (NOS3, eNOS) [5]. Among others, nNOS is mainly expressed in the central and peripheral nervous system, kidney, pancreas, and skeletal muscle [6]. The inducible form of NOS was initially identified as a mediator of innate immunity and macrophages and could be induced in different cell types like vascular smooth muscle cells, renal tubular epithelium, hepatocytes, and mesangial cells [7]. The expression of the eNOS is largely restricted to the vascular endothelial cells and mainly in medium- and large-sized arteries and arterioles [7].

### 1.2. Nitric Oxide and Oxygen

Not only is the production of NO oxygen dependant but also NO plays a very important role in the regulation of O_2_ delivery through vasomotor control locally and cardiovascular and respiratory response centrally. O_2_ is well known for its important function in cellular energy production. O_2_ carrying capacity and saturation of the blood flow are the principle determinants of tissue O_2_ delivery. Therefore, NO plays a major role in regulating vascular tone and organ function in the setting of hypoxia [8]. Paradoxically, hypoxic environment decreases eNOS expression and function which shows us that the view of NO as only a regulator of the vasotonus or blood pressure is too simple. In the last years, the NO signal cascade is discussed as a “sense-and-response” pathway for reduced O_2_ bioavailability through an interaction with the O_2_-sensing pathway (for review see [9]). Another example pointing to the complexity of the role of the L-arginine/NO pathway under hypoxic conditions was shown by Howell et al. [10]. They could demonstrate that supplementation of L-arginine promotes angiogenesis within the gas exchange region of hypoxic lungs and it attenuated the development of pulmonary hypertension in rats in a NO-independent manner [10]. This shows that, beyond the function as a substrate for the NOS, L-arginine seems to have additional proangiogenic properties especially in the pulmonary circulation.

### 1.3. Endogenous NOS Inhibitors in Cardiovascular Disease

**Figure 1:**
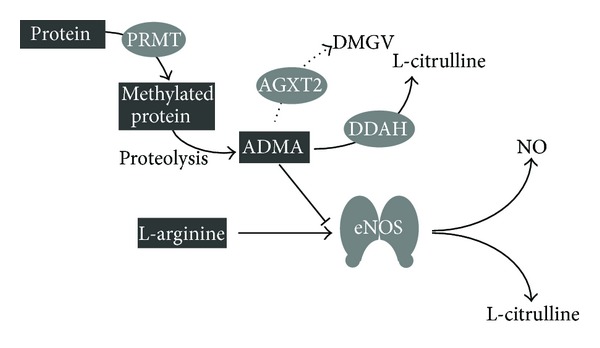
Endothelial L-arginine/NO pathway. L-arginine residues in proteins are methylated by protein-arginine methyltransferases (PRMT), after proteolysis ADMA is released and could replace L-arginine from the binding site at the NOS. ADMA is mainly degraded by dimethylarginine dimethylaminohydrolases (DDAH) to L-citrulline. The degradation of ADMA by alanine-glyoxylate aminotransferase 2 (AGXT-2) to *α*-keto-*δ*-(N(G),N(G)-dimethylguanidino)valeric acid (DMGV) is described as an alternative way which metabolized ADMA only to a very small proportion. This ADMA/AGXT-2 pathway is not object of this review.

N-guanidino-dimethylation of L-arginine residues in proteins by protein-arginine methyltransferases (PRMTs) and subsequent proteolysis lead to the release of free dimethylated L-arginine analogous in the tissue and plasma (Figure 1) [11]. ADMA is known to be an inhibitor of all three isoforms of NOS. It competes with L-arginine for the binding site in the active centre of NOS [12]. Furthermore, ADMA can “uncouple” the NOS by shifting the balance of NO generation to the side of superoxide production. *In vitro* and *in vivo* studies demonstrate that an increase in ADMA could lead to an impaired NO bioavailability as well as an increase in the formation of reactive oxygen species (ROS) [13]. Another dimethylated L-arginine analogue is the symmetric dimethylarginine (SDMA), but its role in the endothelial NO pathway is still unclear. SDMA and ADMA are able to interfere with the substrate availability of NOS by inhibiting the accordant transmembrane cationic amino acid transport (CAT) system of L-arginine, but the IC_50_ values are above the estimated endogenous ADMA and SDMA concentrations [14]. In a large number of prospective clinical studies, ADMA has been characterized as a predictor of major cardiovascular events and mortality in patients with low, medium, and high cardiovascular risk [15, 16]. Some recent studies suggest that SDMA is also associated with cardiovascular events [17, 18] and we have shown that SDMA, but not ADMA, is predictive of all-cause mortality after ischemic stroke [19, 20]. Almost 80% of ADMA is enzymatically hydrolyzed by the dimethylarginine dimethylaminohydrolase (DDAH). DDAH is expressed in two isoforms, DDAH-1 and DDAH-2, which are characterized by distinct tissue distribution, are encoded by different genes, and may exert distinct functional roles [21, 22]. Overexpression of DDAH-1 or DDAH-2 rescues mice from adverse effects of ADMA infusion and improves recovery from vascular damage [23–28]. Transient siRNA-mediated knock-down experiments in rats imply specific functions of DDAH isoforms. Based on these experiments, it appears that DDAH-1 is the dominant form regulating plasma ADMA levels, whereas DDAH-2 appears to be required for actelycholine-dependent vasodilatation [22].

The indirect regulation of the NO bioavailability by varying the ADMA concentrations is discussed as a therapeutic option in various diseases [29]. The regulation of ADMA concentration is possible on different levels. An increase of ADMA formation by enhanced PRMT activity could be seen in the context of different types of human cancer pointing to an decreased NO bioavailability [30]. An enhanced PRMT activity could be also seen in various chronic respiratory diseases leading to the discussion that protein methylation might be a mechanism with therapeutic potential [31]. The effect on NO formation due to increased ADMA concentrations by a reduced ADMA degradation could be recently demonstrated by Ghebremariam and colleagues. They identified a potent DDAH inhibitor which significantly increased intracellular ADMA levels and reduced lipopolysaccharide-induced NO production in endothelial cells [32].

However, it is undisputed that increased concentrations of ADMA and SDMA in tissue and plasma in human as well as in rodents are associated with an unfavorable course of various cardiovascular diseases to an increased mortality. The causing mechanism for ADMA is plausibly the inhibition of the NO production which results in an endothelial dysfunction, but why SDMA is associated with an unfavorable outcome is still unclear. The correlations of ADMA and SDMA with cardiovascular diseases are discussed elsewhere [33–35]. This review will focus especially on the respiratory system and the effects of hypoxia on the endothelial ADMA/NO pathway.

## 2. Clinical Perspective: The ADMA/NO Pathway and Endothelial Function in the Respiratory System—For Better or Worse?

### 2.1. Pulmonary Arterial Hypertension

It is undisputed that in the healthy lung NO plays a key role in maintaining the ventilation/perfusion matching as a response of local hypoxia. In regions of low ventilation the NO levels are low resulting in a vasoconstriction causing the blood flow directed towards well-ventilated regions with high levels of NO to ensure efficient oxygenation of the blood. In patients with pulmonary arterial hypertension (PAH), activity of NOS is reduced compared with those of controls leading to a mismatch in the ventilation/perfusion system [36]. ADMA as a natural occurring inhibitor of NOS is increased in patients with PAH and is associated with unfavorable pulmonary hemodynamics and worse outcome in these patients [37]. The underlying mechanism is a decreased expression and activity of DDAH-2 which was shown in lungs from patients with idiopathic pulmonary arterial hypertension (IPAH) as well as in lungs of Monocrotaline-treated rats [38]. However, an increased vasoconstriction in the pulmonary circulation is only one aspect in the complex pathogenesis of PAH. PAH arises from a combination of pulmonary vasoconstriction, from vascular wall remodeling, from in situ thrombosis, and, in advanced stage of disease, from complex vascular (plexiform) lesion resembling neoangiogenesis within completely obliterated vessels [39, 40]. Besides endothelial injury, invasion of the intima by fibroblast-like cells and enhanced matrix deposition, the proliferation of endothelial cells, are responsible for the intimal changes in the vasculature resulting in hypoxemia what than contributes to the progression of the progression of PAH [41]. NO is a potent stimulator of endothelial cell proliferation, migration, and angiogenesis [42]. Inhibition of NO generation by ADMA in endothelial cells leads to enhanced apoptosis [43]. In the lung phosphodiesterase (PDE) isoenzymes—especially PDE-3 and PDE-4—are important regulators of the cAMP degradation and are upregulated in experimental models of PAH [43]. It has been shown that cAMP-elevating agents enhance EC function, especially angiogenesis [44]. Treatment of endothelial cells with a combined PDE-3/4 inhibitor significantly decreased this ADMA-induced apoptosis by regulating DDAH-2 activity in a cAMP-dependent manner [43].

Drugs targeting the NO pathway are of great interest in the therapy of PAH. Inhaled NO or NO-donors are suitable for short-term use, but due to the development of tolerance, the significant number of nonresponders, and the risk of a rebound effect, NO and NO-donors are not suitable as long-term treatment. Targeting the NO-sGC-cGMP axis downstream of NO seems to be more promising. Inhibiting the degradation of cGMP by inhibiting the PDE-5 has been approved for the treatment of PAH [45–47]. Stimulation of the NO receptor soluble guanylate cyclase (sGC) with Riociguat is another therapeutic strategy acting independently of NO levels [48]. Riociguat has shown promising results in clinical trials and might be available soon [49, 50].

### 2.2. Asthma

Bronchial inflammation, especially in allergic asthma, is triggered by a cascade of proinflammatory mediators including NO [51]. Besides the infiltration and activation of inflammatory cells in the airways, one key pathogenic feature in asthma is the hyperresponsiveness of the airways starting from airway endothelial and smooth muscle cells. Due to an increased iNOS expression in the lung epithelium [52], the expired NO levels are increased in asthmatic patients [53], but in contrast to that the local bioavailability of L-arginine [54] as well as the NO production by the constitutive NOS in smooth muscle cells is reduced [55]. Consistent with this an alteration in L-arginine metabolism especially in the L-arginine degradation by arginase is associated with airflow abnormalities in patients with severe asthma [56]. This mismatch in the L-arginine/NO pathway contributes to the hyperresponsiveness in the airways of asthmatic patients. Looking at the ADMA/NO pathway in these patients, it is clearly shown that ADMA is increased in peripheral compartments (e.g., plasma) as well as locally in lung specimens, sputum, and exhaled breath condensate [57]. The increase in ADMA is often accompanied by a reduced L-arginine bioavailability leading to a decreased L-arginine/ADMA ratio which is proposed to be a novel index reflecting an imbalance in NOS activity caused by an accumulation of ADMA [58]. In a mouse model of allergic asthma, increased ADMA concentrations in the lung caused by a decreased DDAH expression potentiate airway inflammation via modulation of iNOS [59, 60]. This again provides support that the ADMA/DDAH pathway seems to be the key regulator of the L-arginine/NO signaling in a diseased vasculature.

### 2.3. Chronic Obstructive Pulmonary Disease

Approximately 600 million people worldwide are suffering from chronic obstructive lung disease (COPD). COPD is the fourth leading cause of death mainly due to tobacco smoking and must call a global problem. COPD is the only disease whose incidence is increasing constantly. The pathophysiological concept suggests an inflammatory burden and remodeling in the lung leading to the destruction of the elastic architecture of the lung and enlargement of distal air space [61]. In about 30–70% of these patients, the COPD is accompanied by pulmonary hypertension [62]. Pulmonary hypertension is often thought to be a consequence of hypoxic conditions in combination with tobacco smoking in patients with advanced COPD. The impact of a vascular pathology for the pathogenesis is still unresolved. Oxidative and nitrosative stress have been suggested as factors involved in the chronic inflammation and enhanced proliferation processes in the pathogenesis of COPD [63]. In induced sputum samples of unstable COPD patients, an increased number of cells expressing iNOS and nitrotyrosine could be counted [63]. Nitrotyrosine is the reaction product of tyrosine residues and peroxynitrite, which is formed by the reaction of NO and superoxide [51]. In this context, NO derived from iNOS is a key player and is closely linked to the vascular pathology to emphysema development. In an established experimental COPD mouse model, the inhibition of iNOS by L-NIL as well as the genetic depletion of iNOS protected against the development of PH and Emphysema. In this context the iNOS downregulation was associated with a reduced number of proinflammatory cells like granulocytes, macrophages, activated macrophages, and T cells [64]. Interestingly, after full establishment of emphysema, iNOS inhibition was associated with curative restored lung structure and lung function [64]. About the role of the ADMA/NO axis in this context is little known. In exhaled breath condensate of patients with COPD increased ADMA concentrations have been seen [65]. The NOS inhibitory capacity of ADMA leads to the assumption of a regulatory function of ADMA regarding the NO bioavailability in COPD. But this needs to be verified.

### 2.4. Idiopathic Pulmonary Fibrosis

Idiopathic pulmonary fibrosis (IPF) characterized by an injury of alveolar epithelium, alveolar inflammation, and increased proliferation of fibroblast is the most common and aggressive form of lung fibrosis. In the process, IPF could result in a progressive loss of alveolar capillaries and lung architecture, which dramatically affects sufficient oxygenation and is therefore associated with high morbidity and mortality [66]. IPF is not typically defined as a vascular disease. However, the final stage of IPF resulted in hypoxia which could then effect secondarily the vascular system. But up to now little is known about the pathomechanisms and the involvement of the vascular system especially of the endothelial system. However, what is certain is the involvement of the inducible NOS in the pathophysiology of IPF. Lung protein levels of iNOS were three times higher in patients with IPF compared with control donors and were observed close to fibrotic scares, thickened septa, and fibroblast foci. Interestingly, this iNOS expression was accompanied by an increased colocalized expression of DDAH-2 [67]. This colocalization suggests an ADMA-related regulation of the iNOS. In mice with bleomycin-induced fibrosis, an increased immunoreactivity of DDAH-1 and DDAH-2 was detected in the endothelium, inflammatory cells, fibroblasts, airway epithelial cells, and alveolar epithelial cells. This increase of DDAH was associated with decreased ADMA levels. Surprisingly DDAH inhibition by the L-291 suppressed the abnormal proliferation of alveolar epithelial cells in IPF and induced apoptosis in an ADMA-dependent manner. Additionally, DDAH inhibition as well as iNOS inhibition reduced collagen production by fibroblasts and improved lung function in bleomycin-treated mice [67]. This example of IPF demonstrated the controversy of increased ADMA concentration in the diseased respiratory vascular system.

### 2.5. Lung Cancer

Intensive investigation has been conducted on the role of the NO pathway in cancer. NO plays a role in cellular proliferation, migration, induction of epithelial-mesenchymal transition, angiogenesis, and apoptosis of cancer cells. Increased NO concentration can be detected in the microenvironment of many solid cancers. However, the role of NO appears ambiguous and may indicate a biphasic nature of NO-mediated cellular effects depending on its concentration at the site of cancer cells, the chemical redox environment, and the duration of NO exposure; that is, NO can act pro- and antitumorigenic (reviewed by [68]). In non-small-cell lung cancer, increased expression of iNOS has been observed in tumor tissue, and patients exhale elevated NO levels [69]. Expression of iNOS contributes to the urethane-induced and to genetically, Kirsten rat sarcoma viral oncogene homolog (KRAS) mutation-induced lung carcinogenesis whereas inhibition of iNOS reduced carcinogenesis in animal cancer models [70, 71]. Hypoxia occurs within growing and expanding tumor tissue and may drive (neo-)angiogenesis, and cause, if not caught up by novel vessel generation, tumor necrosis due to lacking tumor cell nutrition and oxygenation. The histopathological extend of intratumoral necrosis is associated with unfavorable prognosis in lung cancer and other entities such as colorectal cancer [72, 73]. Hypoxia stimulates iNOS expression and NO production and hereby may contribute to tumor blood supply [74]. Interestingly, intravenous administration of the NOS inhibitor N^*Ω*^-nitro-L-arginine (L-NNA) reduced the tumor blood supply in patients with non-small-cell lung cancer providing the early clinical evidence that inhibition of NOS has antivascular activity in cancer [75]. Preclinically, privation of blood flow caused by NOS inhibition can be restored by administration of L-arginine underlying the NO dependence of cancerous vascularity [76]. A small study showed that plasmatic levels of the intrinsic NOS inhibitor ADMA are increased in patients with small and non-small-cell lung cancer without concomitant cardiovascular diseases as compared to healthy subjects [30]. In this study comparable elevation of ADMA has been observed in other epithelial cancers such as gastric and breast cancer indicating a vascular response involving the ADMA/NO pathway in cancer patients [30]. The biological significance and prognostic role of ADMA in lung cancer are yet unknown.

### 2.6. Obstructive Sleep Apnea Syndrome

Obstructive sleep apnea syndrome (OSAS) is defined by the presence of symptoms such as daytime sleepiness in conjunction with a significant quantity of obstructive events occurring during sleep. The registration of ventilator event includes episodes of apneas and hypopneas and increased upper airway resistance [77]. OSAS has been found to be an independent risk factor for cardiovascular events [78]. Episodes of desaturation-reoxygenation during night are a typical pattern. This sequence, defining intermittent hypoxia, causes the generation of oxidative stress such as production of ROS which contributes to systemic inflammation found in these patients [77]. Oxidative stress and inflammatory process such as increased leukocyte adhesion via expression of adhesion molecules promote endothelial damage and dysfunction [79]. Thus, impaired endothelium-dependent vasodilatation is typically found in patients with OSAS [80] and can partly be reversed by continuous positive airway pressure (CPAP) therapy indicating a crucial pathophysiological link between the endothelial dysfunction and intermittent hypoxemia in OSAS [81]. The NO metabolism has been strongly implicated in this relationship. Levels of circulating NO measured as serum nitrites and nitrates were significantly lower in patients with OSAS and correlated negatively with parameters of disease severity in these patients [82]. Furthermore, plasma ADMA levels are elevated in patients with OSAS irrespective of the presence of further cardiovascular risk factor [83]. Following CPAP therapy with significant reduction of intermittent episodes of hypoxemia, levels of NO and ADMA can be restored [82, 84].

## 3. Clinical Perspective: Hypoxia and Endothelial Function in the Respiratory System

Alveolar hypoxia redirects the capillary blood flow to areas of higher oxygen availability by hypoxia-induced vasoconstriction of pulmonary arterial vessels [85, 86]. This mechanism accounts for a sufficient maintenance of blood oxygenation. Cells of the precapillary smooth muscle layer of vessels located at the entrance of the acinus are thought to be the sensor and effector cell-type in this mechanism in a calcium-dependent manner (reviewed by [87]). However, hypoxia-induced pulmonary vasoconstriction can be abolished by denudation of the endothelial layer as demonstrated in porcine small pulmonary vessels [88]. This observation underlies the complexity of the intercellular regulatory network in response to acute hypoxia. Mediators derived from the pulmonary arterial endothelial cells critically regulate the vascular tone in response to hypoxia (reviewed by [89]). Chronic respiratory diseases such as COPD or fibrosis are associated with sustained systemic hypoxemia by altered gas-exchange due to increased diffusion distance, poor ventilation, or loss of alveolar structures. In contrast to acute or subacute hypoxia, with sustained hypoxia, a temporary vasodilatation has been described, followed by a secondary vasoconstrictor response [87]. The response of endothelial cells differs from that followed by acute hypoxia, while under acute hypoxic condition pulmonary endothelial cells have been shown to slow down their cell cycle progression (but did not arrest), and under chronic hypoxic conditions pulmonary endothelial cells exhibited enhanced proliferation [90, 91]. On cellular level, exposure to chronic hypoxia leads to progressive pulmonary vascular remodeling associated with increased vascular resistance and development of a pulmonary hypertension phenotype [92]. In humans vascular remodeling consisting of thickened pulmonary vessels and resulting in elevated pulmonary arterial resistance is evident under chronic hypoxic conditions in high altitude [93]. In patients with underlying hypoxia-related respiratory disease, such as COPD, mild elevation of pulmonary arterial pressure is frequent and associated with unfavorable prognosis regarding all-cause mortality [62]. However, in this relationship clinical data revealed no clear correlation between grade of hypoxemia and magnitude of pulmonary hypertension indicating further mechanism, in addition to hypoxemia, that leads to vascular remodeling [94]. A possible additional aspect might be the link between chronic lung inflammation, systemic inflammation, and as a result vascular inflammation which leads to dysfunctional endothelial cells and causes cardiovascular morbidity [95].

## 4. Hypoxia as a Mediator of ADMA/NO-Related Endothelial Dysfunction: Little Is Known

Clinical as well as experimental data clearly show that the dysregulation of the ADMA/NO pathway plays a crucial role in the development and/or progression of hypoxia associated chronic respiratory diseases. But the underlying molecular mechanisms can be varied and are not really clear. Hypoxia as a cause for vascular changes or hypoxia as a consequence of chronic respiratory diseases seems to play a major role in the regulation of the ADMA/NO pathway.

**Figure 2:**
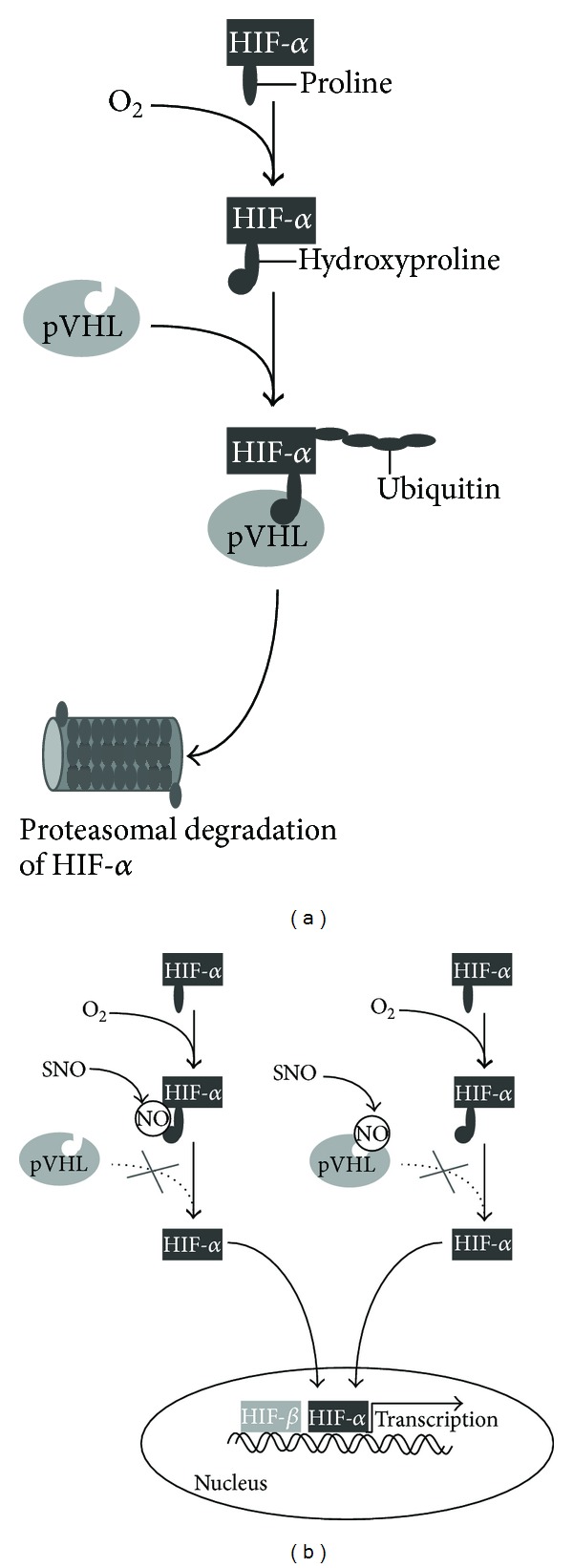
S-nitrosylation of HIF-*α* and pVHL. (a) In the presence of oxygen proline, residues of HIF-*α* are hydroxylated. This leads to the polyubiquitination of the pVHL-HIF-*α* complex resulting in the proteasomal degradation of HIF-*α*. (b) S-nitrosylation of HIF-*α* prevents the binding of pVHL and thereby the polyubiquitination of HIF-*α*. S-nitrosylation of pVHL also inhibits the ability to mediate the polyubiquitination of HIF-*α*. In both cases HIF-*α* is not degraded but translocated into the nucleus, dimerizes with the HIF-*β* subunit, and induces the transcriptional activation of target genes.

Acute and chronic changes in oxygen levels lead to the activation of comprehensive sense and response mechanisms in the whole organism or locally in different organs and tissues. The heart of these response mechanisms is the hypoxia-inducible factor (HIF) which consists of a HIF-1 *α*-subunit (HIF-1*α*) and a nuclear *β*-subunit (HIF-1*β*) [96]. Under normoxic conditions, HIF-1*α* is bound by Von Hippel-Lindau protein (pVHL) [97]. The binding of pVHL is dependent on the hydroxylation of a specific proline residue in HIF-1*α* by the oxygen-dependent prolyl hydroxylase (PHD) 2. PHD-2 uses O_2_ as a substrate and thus PHD-2 activity is inhibited under hypoxic conditions [98]. In this bound and inactive form HIF-1*α* is proteasomal degraded by an ubiquitin ligase (Figure 2(a)) [99]. HIF-2*α*, a paralog of HIF-1*α*, is found in vertebrates and is also regulated by prolyl hydroxylation. HIF-2*α* also dimerizes with HIF-1*β* and plays an important role in erythropoiesis, vascularization, and pulmonary development [100]. The pathological consequences of HIF-1 dysregulation in chronic diseases include a wide range of both protective and pathogenic responses. Diseases in which HIF-1 mediates protective responses are coronary artery disease [101], peripheral arterial disease [102], Colitis [103], and organ transplant rejection [104]. In cancer [105] and chronic respiratory diseases like PAH [106] and OSAS [107] HIF-1 activity contributes to the pathogenesis of the disease.

Interestingly, there seems to be a mutual regulation of NO signaling and hypoxic HIF signaling. One key mechanism by which NO regulates cellular targets or hypoxia signaling is S-nitrosylation [108, 109]. Components of the HIF-1*α* signaling are targets for S-nitrosylation resulting in a stabilization of HIF-1*α* under normoxic conditions [109, 110]. Direct S-nitrosylation of the Cys-533 of HIF-1*α* prevents the binding of pVHL and the following polyubiquitination (Figure 2(b)). S-nitrosylation of Cys-162 of pVHL prevents the binding of pVHL to HIF-1*α* (Figure 2(b)) and also inhibits its ability to mediate the polyubiquitination of HIF-1*α* [110]. Additionally, it has been shown that under hypoxic conditions different concentrations of NO—as an inhibitor of the mitochondrial cytochrome c oxidase—have different effects on HIF-*α* stabilization. Concentrations of NO < 400 nM resulted in a decrease of HIF-*α* stabilization whereas NO concentrations of > 1 *μ*M caused a stabilization of HIF-*α* [111]. This could be explained by an increase in oxygen-independent PHD-dependent degradation of HIF-*α* [112].

Additionally NO can also inhibit PHD activity. It competes with O_2_ for Fe^2+^ at the catalytic domain of PHDs and supports the stability of HF-1*α* [113]. The molecular mechanisms by which hypoxia could regulate NO production in the endothelium are diverse. They range from transcriptional and epigenetic modifications to posttranscriptional and posttranslational modifications of the NOS (reviewed by [9]). This direct interaction between NO signaling and hypoxia signaling is one regulatory possibility. In pulmonary endothelial cells hypoxia can also inhibit the substrate availability of the eNOS by inhibiting the transport of L-arginine into the endothelial cell [114]. Additionally, we and others proposed that DDAH is the key determinant of intracellular ADMA concentrations and that the regulation of DDAH could therefore modulate NO bioavailability indirectly [29, 32, 115]. In 2001 Murray-Rust et al. [116] identified a Cys-His-Glu catalytic triad and Leiper et al. showed in 2002 that the presence of the reactive cysteine residue is directly regulated by NO mediated reversible S-nitrosylation [117]. So, under circumstances when NO generation increases, NO mediated S-nitrosylation inhibited DDAH activity, which leads to accumulation of ADMA and inhibition of NOS. It is conceivable that this cycle is active in all normoxic states of a modified NO generation as well as in the status of hypoxia. It recently has been shown that DDAH-1 overexpression in mice decreased sustained hypoxia-induced pulmonary vasoconstriction but did not alter the vascular response to acute or chronic hypoxia. This effect of DDAH-1 overexpression could be partly explained by an ADMA-induced inhibition of the NO pathway [118]. This study in combination with the clinical data on DDAH in chronic respiratory diseases supports the fact that hypoxia mediate DDAH activity, but the molecular mechanisms behind this hypoxia-mediated regulation are still lacking. It is generally accepted that hypoxia is associated with a high burden of ROS. One possible link between hypoxia and DDAH regulation might be the hypoxia-induced formation of ROS. Ito et al. showed that DDAH activity is inhibited by oxLDL in endothelial cells [119] leading to increased ADMA concentrations. In addition, the incubation of cultured endothelial cells with glucose resulted in an impaired DDAH activity and subsequently increased ADMA levels. This glucose-mediated effect was inhibited by antioxidants [120]. The impact of oxidative stress and DDAH is reviewed in detail by Sydow and Münzel 2003 [121]. The regulation of DDAH by ROS occurs mainly at the level of enzyme activity. There are some studies describing the genetic regulatory promoter regions of DDAH-1 and DDAH-2 and the mediated effects through activation of these regions under normoxic conditions. Both promoter regions of DDAH-1 and DDAH-2 contain a sterol response element which could bind the statin-induced transcription factor sterol response element binding protein (SREBP). Ivashchenko et al. demonstrated a reciprocal regulation by SREBP-2 and SREBP-1 of DDAH-1 and could therefore explain the positive ADMA-lowering effect by simvastatin which might therefore contribute to the vasculoprotective effect of statins [122]. Hasegawa et al. could identify a specific protein 1 (SP1) binding site in the DDAH-2 promoter which is responsible for the DDAH-induced expression and secretion of the vascular endothelial growth factor (VEGF), resulting in an increase in proliferation and migration of endothelial cells. This effect is not dependent on the ADMA/NO pathway [123]. Jung et al. showed that Vaspin, an adipocitokine expressed in the visceral adipose tissue, mediated its antiatherogenic effect by a STAT-3 activation of the DDAH-2 promoter resulting in a decreased ADMA-induced inhibition of eNOS [124]. Another promoter modification of DDAH-2 is described by Eikelboom et al. They showed that Hyperhomocysteinemia, which is also associated with an increased risk for cardiovascular diseases [125], leads to a dose-dependent hypermethylation of the CpG island in the DDAH-2 promoter region. This hypermethylation was associated with an impaired mRNA expression of DDAH-2 [126]. These studies lead to the suggestion that there might be also a genetic regulation of the ADMA/DDAH pathway via hypoxia but the molecular crosslink between DDAH and hypoxia signaling besides ROS is not known so far.

## 5. The ADMA/NO Pathway: Therapeutic Potential in Respiratory Diseases?

The direct, therapeutic regulation of NOS in the field of cardiovascular diseases has been long discussed as a novel therapeutic strategy. Especially in chronic respiratory lung diseases, both beneficial and deleterious effects of NO have been shown in the airways. But up to now there is no potential direct NOS regulator for the treatment of cardiovascular diseases including chronic respiratory diseases available. The therapeutic regulation of ADMA via DDAH might be another possible mechanism to regulate NOS and therefore NO bioavailability indirectly. However, the therapeutic potential of DDAH is controversial. From many clinical studies we know that increased ADMA concentrations promote an endothelial dysfunction and remodeling processes in the lung. There is evidence that ADMA contributes to the pathogenesis of various diseases and that the inhibition of its degradation has protective properties. The positive impact of DDAH inhibition and therefore increased ADMA concentration has been already discussed in the setting of idiopathic pulmonary fibrosis [32, 127] and Endotoxic shock [128]. It is also conceivable that ADMA is anyhow involved in the local hypoxia-induced pulmonary vasoconstriction resulting in the maintenance of the ventilation/perfusion match in the lung. However, the clinical relevance of a regulation of the endothelial ADMA/NO pathway needs to be evaluated and the development of DDAH regulators is a promising approach as new therapeutic targets in some chronic respiratory diseases.

## Abbreviations

ADMA: Asymmetric dimethylarginine
AGXT: Alanine-glyoxylate aminotransferase
CAT: Cationic amino acid transporter
cAMP: Cyclic adenosine monophosphate
cGMP: Cyclic guanosine monophosphate
COPD: Chronic obstructive pulmonary disease
CPAP: Continuous positive airway pressure
DDAH: Dimethylarginine dimethylaminohydrolase
eNOS: Endothelial nitric oxide synthase
HIF: Hypoxia-induced factor
iNOS: Inducible nitric oxide synthase
IPAH: Idiopathic pulmonary arterial hypertension
IPF: Idiopathic pulmonary fibrosis
KRAS: Kirsten rat sarcoma viral oncogene homolog
L-NNA: N-nitro-L-arginine
nNOS: Neuronal nitric oxide synthase
NO: Nitric oxide
NOS: Nitric oxide synthase
OSAS: Obstructive sleep apnea syndrome
PAH: Pulmonary arterial hypertension
PDE: Phosphodiesterase
PHD: Prolyl hydroxylase
ROS: Reactive oxygen species
SDMA: Symmetric dimethylarginine
sGC: Soluble guanylate cyclase
SREBP: Statin-induced transcription factor sterol response element binding protein
SP1: Specific protein 1
STAT: Signal transducer and activator of transcription 3 (acute-phase response factor)
VEGF: Vascular endothelial growth factor
pVHL: Protein von Hippel-Lindau.
